# Assessment of Barriers and Facilitators to the Delivery of Care for Noncommunicable Diseases by Nonphysician Health Workers in Low- and Middle-Income Countries: A Systematic Review and Qualitative Analysis

**DOI:** 10.1001/jamanetworkopen.2019.16545

**Published:** 2019-12-02

**Authors:** David J. Heller, Anirudh Kumar, Sandeep P. Kishore, Carol R. Horowitz, Rohina Joshi, Rajesh Vedanthan

**Affiliations:** 1 Arnhold Institute for Global Health, Icahn School of Medicine at Mount Sinai, New York, New York; 2 Department of Medicine, New York University School of Medicine, New York; 3 Department of Medicine, Brigham and Women’s Hospital, Boston, Massachusetts; 4 The George Institute for Global Health, University of New South Wales, Sydney, New South Wales, Australia; 5 The George Institute for Global Health, New Delhi, India; 6 Faculty of Medicine, University of Sydney, Sydney, New South Wales, Australia; 7 Department of Population Health, New York University School of Medicine, New York

## Abstract

**Importance:**

Cardiovascular disease, cancer, and other noncommunicable diseases (NCDs) are the leading causes of mortality in low- and middle-income countries. Previous studies show that nonphysician health workers (NPHWs), including nurses and volunteers, can provide effective diagnosis and treatment of NCDs. However, the factors that facilitate and impair these programs are incompletely understood.

**Objective:**

To identify health system barriers to and facilitators of NPHW-led care for NCDs in low- and middle-income countries.

**Data Sources:**

All systematic reviews in PubMed published by May 1, 2018.

**Study Selection:**

The search terms used for this analysis included “task shifting” and “non-physician clinician.” Only reviews of NPHW care that occurred entirely or mostly in low- and middle-income countries and focused entirely or mostly on NCDs were included. All studies cited within each systematic review that cited health system barriers to and facilitators of NPHW care were reviewed.

**Data Extraction and Synthesis:**

Assessment of study eligibility was performed by 1 reviewer and rechecked by another. The 2 reviewers extracted all data. Reviews were performed from November 2017 to July 2018. All analyses were descriptive.

**Main Outcomes and Measures:**

All barriers and facilitators mentioned in all studies were tallied and sorted according to the World Health Organization’s 6 building blocks for health systems.

**Results:**

This systematic review and qualitative analysis identified 15 review articles, which cited 156 studies, of which 71 referenced barriers to and facilitators of care. The results suggest 6 key lessons: (1) select qualified NPHWs embedded within the community they serve; (2) provide detailed, ongoing training and supervision; (3) authorize NPHWs to prescribe medication and render autonomous care; (4) equip NPHWs with reliable systems to track patient data; (5) furnish NPHWs consistently with medications and supplies; and (6) compensate NPHWs adequately commensurate with their roles.

**Conclusions and Relevance:**

Although the health system barriers to NPHW screening, treatment, and control of NCDs and their risk factors are numerous and complex, a diverse set of care models has demonstrated strategies to address nearly all of these challenges. These facilitating approaches—which relate chiefly to strong, consistent NPHW training, guidance, and logistical support—generate a blueprint for the creation and scale-up of such programs adaptable across multiple chronic diseases, including in high-income countries.

## Key Points

**Question:**

What are the health system factors that support or impair the ability of nonphysician health workers to treat noncommunicable diseases in low- and middle-income countries?

**Findings:**

This systematic review and qualitative analysis examined 15 systematic reviews, encompassing 71 studies. These studies consistently demonstrated 6 key lessons of successful care by nonphysician health workers: careful staff recruitment, detailed training, authorization to provide autonomous care, adequate medications and supplies, reliable data systems, and fair, performance-based compensation.

**Meaning:**

Effective, scalable care for noncommunicable diseases led by nonphysicians is feasible in diverse low-resource settings but requires several common, key implementation steps.

## Teaser

This systematic review and qualitative analysis examines the health system barriers to and facilitators of care for noncommunicable diseases by nonphysician health workers in low- and middle-income countries.

## Introduction

Noncommunicable diseases (NCDs) are the leading causes of premature death worldwide.^1^ Noncommunicable diseases are increasingly prevalent in low- and middle-income countries (LMICs), especially their most vulnerable communities,^2,3^ where human resources for health are severely limited.^4^ More than one-half of LMICs have fewer than 1 physician per 1000 people,^5^ which is the minimal threshold advised by the World Health Organization (WHO), and in many LMICs, less than one-quarter of physicians practice in rural areas where one-half of the population lives.^6^

Fortunately, evidence demonstrates that nonphysician health workers (NPHWs) (ie, persons without a medical doctorate degree who render health care)^7,8,9,10^ can render multiple aspects of care presumed to require a physician.^7,8^ Models of care that employ NPHWs—including health professionals (eg, nurses) and laypeople (eg, community health workers [CHWs])—have successfully treated many causes of death and disability, especially maternal-child mortality and HIV/AIDS.^7,8,9^

Models that successfully leverage NPHWs for NCD control also show promise.^10^ Pilot studies^11,12,13^ demonstrate that NPHWs can accurately perform cardiovascular risk screening to identify high-risk patients and counsel them on behavior change. In addition, NPHWs can screen for and treat risk factors associated with cardiovascular disease, such as depression,^14,15^ diabetes,^16^ and hypertension,^16^ including by prescribing medication, and can track and improve patients’ adherence to these therapies. Nonphysician health workers can also screen for cancers^17,18^ and treat epilepsy^19^ and asthma,^20^ among other chronic diseases.

However, the rapid increase of NCDs in LMICs requires the scale-up of these programs into global initiatives,^21,22^ as recent United Nations summits have demanded.^23,24^ This demand poses a problem: understanding which elements of health systems (eg, governance and delivery structures) support or hinder NPHWs in the care cascade.^10^

## Methods

**Figure 1.**
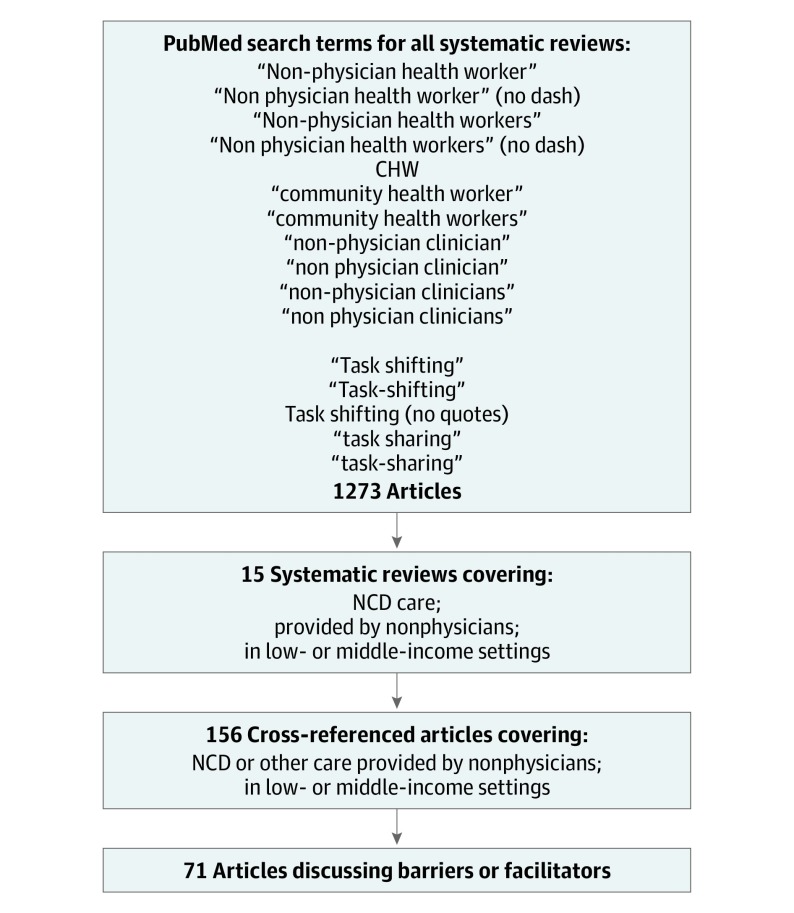
Process for Identifying Relevant Articles Flowchart shows search terms and criteria used to identify relevant articles for analysis. CHW indicates community health worker; NCD, noncommunicable disease.

We undertook a systematic review and qualitative analysis to identify and analyze health system barriers to and facilitators of NPHW-led care for NCDs, as detailed within systematic reviews of these interventions in LMICs. We defined a health system according to the WHO Health Systems Framework’s 6 building blocks^25,26^: service delivery, health workforce, governance, information systems, medication access, and financing. This descriptive analysis of these heterogeneous interventions did not compile quantitative outcomes, nor did it evaluate a hypothesis. We conducted and reported this review per the Meta-analysis of Observational Studies in Epidemiology (MOOSE) reporting guideline,^27^ and we display studies identified, screened, reviewed, and included per the Preferred Reporting Items for Systematic Reviews and Meta-analyses (PRISMA) reporting guideline^28^ in Figure 1. We did not seek formal review from an institutional review board because we limited our study to published information and did not engage with any human subjects.

### Objectives of the Review

We aimed to identify all systematic reviews of initiatives leveraging NPHWs for the treatment of NCDs in LMICs. We defined an NCD as any chronic disease independent of infection, but included long-term conditions caused by infection, such as poststreptococcal rheumatic heart disease.^29^ We defined LMICs according to 2016 World Bank criteria.^30^ We considered a study to be leveraging NPHWs if these staff engaged in clinical decision-making for patient care, regardless of physician supervision. This approach includes models for both task-shifting completely from physicians to NPHWs and task-sharing, in which NPHWs assume care under physician oversight.^10^

### Literature Searching

We began our review with 6 systematic reviews already known to us that addressed all of the aforementioned subjects (ie, sentinel reviews). One reviewer (D.J.H.), a clinician-investigator trained in implementation science and public health, searched PubMed from its inception to May 1, 2018, for all systematic reviews that examined NCD care performed by NPHWs in LMICs. Our search terms, developed in consultation with a librarian specializing in advanced search techniques,^31^ included “non-physician health worker(s),” “task-shifting,” “task-sharing,” “community health worker,” “CHW,” or “non-physician clinician(s)” (Figure 1). We did not expressly search for terms such as “nurse” or “health system,” nor did we exclude them. We excluded all articles that did not focus on NCD care, were not conducted entirely or primarily in LMICs, or were not systematic reviews. A second reviewer (R.J.), a clinician-investigator with expertise in systematic reviews,^10^ repeated the first reviewer’s search independently. We did not use search software or manual searching, nor did we search databases other than PubMed. Both searches agreed completely and identified all 6 aforementioned reviews, as well as 9 others, all written in English. We quantified the rigor of these 15 reviews using the A Measurement Tool to Assess Systematic Reviews–2 tool, which is designed to evaluate systematic reviews of health care interventions.^32^

### Study Eligibility and Data Extraction

Using review methods derived from Green et al,^33^ Iwelunmor et al,^34^ and Popay et al,^35^ 2 coauthors (D.J.H. and A.K., who was then a medical student with public health expertise) reviewed each of these systematic reviews in detail. Each reviewer identified every article referenced within each document that concerned NPHW care for NCDs, referenced barriers to and facilitators of NPHW care (for NCDs or other conditions), was a systematic review, or was referenced in 2 or more systematic reviews. This approach intended to identify any barriers and facilitators of NPHW care relevant to NCD control, but also any systematic reviews that referenced the same but that we had overlooked. The reviewer then examined the abstract of each such article to determine whether it appeared to examine health system issues affecting NPHW-led NCD care. We read each article that explored these issues and identified all concepts related to health systems. We did not contact authors for further details and limited our review to articles published in English. We performed this review from November 2017 to July 2018. Each coauthor who reviewed each article classified the NPHW barriers and facilitators using the WHO’s building block framework,^25,26^ given its extensive prior use and validation in health systems research. The 2 coauthors (D.J.H. and A.K.) reviewed each other’s classification list for agreement. When they could not reach consensus, they consulted a third coauthor (R.J.) for a final decision.

### Categorization of Study Outcomes

After compiling the classification list of health system concepts, we tabulated each documented barrier or facilitator in each reviewed study, as classified by D.J.H. and A.K. Because the study outcomes were heterogeneous, and because our objective was to review qualitative health system factors within these studies rather than those outcomes, we did not perform any meta-analyses or other quantitative analyses, nor did we quantify the quality of these studies apart from the A Measurement Tool to Assess Systematic Reviews calculation.^32^ After compiling all barriers and facilitators, D.J.H. and A.K. qualitatively reviewed the results for key themes, with feedback from R.J. and the other coauthors. Our goal was to identify actionable elements of an NPHW care intervention (eg, how staff are recruited, supervised, or compensated) that appeared to influence the success or failure of the program in delivering care. We used grounded theory—that is, the iterative review of data to code barriers and facilitators and then sort them into concepts—given that our goal was to describe data rather than test a hypothesis.^36^ The report follows the Standards for Reporting Qualitative Research (SRQR) reporting guideline.^37^

### Statistical Analysis

We did not undertake any statistical analysis apart from the tally of barriers and facilitators.

## Results

Our PubMed search yielded 1273 publications. The search terms are shown in the eAppendix in the Supplement. Among these results, we identified 15 systematic reviews^9,10,38,39,40,41,42,43,44,45,46,47,48,49,50^ that focused on NCDs and significantly involved LMICs (Table 1). These documents referenced a total of 156 unique articles. Of these, we identified 71 unique articles that met further review criteria defined in the Methods section (Figure 1). We summarize this content in Table 2, organizing content by key measures of health care performance: quality, access, safety, and coverage.^51^

**Table 1.** Characteristics of Key Articles Used in Review

Study	Type	Disease or Condition Covered	Scope	Studies Included, No. (Total Cited, No.)	WHO Building Blocks Addressed	Types of NPHW Participants	Total Study Participants, No.
Mutamba et al,^42^ 2013	Systematic review	Mental, neurological, and substance abuse disorders	All studies comparing lay community health workers community-level care for these diseases to a control in LMICs	5 (15)	Service delivery, health workforce	Lay community health workers	15 039 (7900 intervention; 7139 control)
Joshi et al,^10^ 2014	Systematic review	All NCDs	All peer-reviewed, English language articles up to 2013 that discuss task-shifting of NCDs to NPHWs	16 (22)	Health workforce, medication access, governance	Nurses or laypersons without medical training	Not provided
Ogedegbe et al,^45^ 2014	Systematic review	CVD	All peer-reviewed, English-language randomized clinical trials up to 2013 to evaluate task-shifting for CVD management in LMICs	2 (3)	Service delivery, health workforce, information systems, governance	Nonphysician clinicians involved in treatment or risk management	3002
Khetan et al,^40^ 2017	Systematic review	CVD	All articles from 1990-2015 involving CHWs for CVD (no other NCDs; no other NPHWs); not limited to randomized clinical trials.	8 (11)	Service delivery, health workforce	CHWs (persons trained in intervention but without formal health training)	78 524
Jeet et al,^49^ 2017	Systematic review	All NCDs (apart from mental health)	All randomized clinical trials from 2000-2015 involving CHWs for NCDs (no other trial types; no other NPHWs)	5 (16)	NA	CHWs, but these included nurses and “health promoters” among many other NPHWs	6621 (Diastolic blood pressure); 6782 (systolic blood pressure); 1342 (diabetes) 7302 (tobacco use) inter alia
Schneider et al,^9^ 2016	Scoping review	All diseases	All articles from 2005-2014 that described an LMIC CHW intervention, regardless of condition	11 (678)	NA	CHWs (lacking formal nursing or medical training)	Not provided
Padmanathan et al,^41^ 2013	Systematic review	Mental illness	All English-language peer-reviewed and gray literature (any study design) on feasibility and acceptability of task-sharing for mental health care in LMICs	8 (21)	Service delivery, health workforce	Any nonspecialist clinician (including nurses, medical officers, and CHWs)	>1116 (Data incomplete)
Abdel-All et al,^44^ 2017	Systematic review	CVD	All peer-reviewed studies published until December 2016 regarding training of CHWs for prevention or control of CVD (and/or risk factors) in LMICs	2 (8)	Health workforce	CHWs (from community; usually lack formal training)	722
Seidman et al,^48^ 2017	Systematic review	All diseases	All literature regarding the cost-effectiveness of nonphysicians for care provision in LMICs (for NCD and non-NCD care)	2 (34)	NA	Any less-specialized health worker (including assistant medical officers)	Not provided
Chowdhary et al,^43^ 2014	Systematic review	Perinatal depression	All literature regarding nonspecialist (including generalist physician) perinatal depression care in LMICs	2 (9)	Service delivery, health workforce	Nonspecialist health workers including nurses, CHWs, mothers	14 555 (7526 Intervention; 7029 control)
Barnett et al,^39^ 2017	Systematic review	Mental illness	All literature regarding CHW (not other NPHWs) care for mental health care in LMIC and high-income countries	9 (39)	Service delivery	CHWs (interventionists without mental health training and from community)	10 199
Hill et al,^47^ 2017	Systematic review	Diabetes	All literature regarding use of CHWs (not other NPHWs) care for diabetes prevention (not treatment) in LMICs and high-income countries	1 (30)	NA	Lay CHWs (nonprofessionals recruited usually from community served)	5834 (Data incomplete)
Alaofè et al,^50^ 2017	Systematic review	Diabetes	All literature regarding use of CHWs (not other NPHWs) care for diabetes prevention and treatment in LMICs	5 (10)	NA	CHWs (community members without formal health training)	69 998
Gatuguta et al,^38^ 2017	Systematic review	Sexual violence, trauma	All literature regarding use of CHWs to treat survivors of sexual violence in LMICs and high-income countries	2 (7)	Health workforce	CHWs (community members without formal health training)	961 (Data incomplete)
Javadi et al,^46^ 2017	Systematic review	Mental illness	All literature regarding nonphysician task-shifting for mental health care in LMICs	23 (30)	NA	Laypersons with minimal mental health training	701 864 (Data incomplete)

Abbreviations: CHW, community health worker; CVD, cardiovascular disease; LMIC, low- and middle-income country; NA, not applicable; NCD, noncommunicable disease; NPHW, nonphysician health worker; WHO, World Health Organization.

**Table 2.** Key Barriers and Facilitators to NPHW Care for Noncommunicable Diseases

Building Block	Facilitators	Barriers	Key Themes	Key Conclusions	Care Aspects: Access, Coverage, Quality, Safety
Service delivery	Home-based or local care; clinician cultural sensitivity; integration of multiple conditions; consistent protocols for patient tracking	Patient education without other care provision; limited patient health literacy; patient transport and safety barriers to accessing care; too few auxiliary and supervisory staff; unclear NPHW roles	Logistics; infrastructure; cultural interaction or stigma	Clinicians benefit from close proximity to the community they serve (home visits or local clinics). Culturally sensitive, locally understandable messages are crucial. Adequate numbers of primary and backup clinicians matter. A clear scope of NPHW care is helpful. Facility-based referral is critical for complex cases and NPHW confidence.	Access: physical and cultural proximity to patients is crucial. Coverage: greater quantity, length, and scope of visits boosts coverage. Quality: protocols for what care is covered, and how patients are tracked, ensure consistency. Safety: patient (and sometimes clinician) safety sometimes at risk in accessing care.
Health workforce	Frequent, intensive training; close supervision; specific care delivery algorithms; integration of role with other clinicians	Delays in training; poor staff retention; lack of clear protocols; excessive workload; lack of oversight; limited NPHW literacy	Training; role and expectation; oversight	Clinicians require rigorous, clear, continuous training. Protocol-based workflow that is straightforward and reasonable in expectation. Oversight and backup by other clinicians is crucial. Careful selection and incentive structure may help locate, retain strong clinicians.	Access: poor staff retention impairs patients’ access. Coverage: intensive training boosts breadth of conditions treated. Quality: checklists and algorithms for care boost delivery standards. Safety: close oversight of NPHWs protects patient safety and may prevent errors.
Governance	Authorization for NPHWs to prescribe medication; integration with other staff roles; engagement of program with local authorities	Lack of authority to prescribe medication; no policies recognizing NPHW roles; skepticism of NPHW care capacity; political upheaval	Political engagement; codification of NPHW role	Policy makers should recognize the evidence base for NPHW care and define their roles accordingly. NPHWs should have care authority commensurate to the evidence base. Roles of NPHWs and other clinicians should be clearly defined relative to other cadres. Programs should promote stable engagement with communities.	Access: engaging community leaders makes patients aware of available programs. Coverage: ability for NPHWs to give medication improves breadth of conditions treated. Quality: close access to supervisory staff boosts quality of care delivery. Safety: clear roles for NPHWs, other clinicians promote safe scope of practice commensurate with experience.
Information systems	Electronic or paper record systems; written patient transfer notes; patient appointment calls or reminder letters; telemedicine consultation mechanisms	Absent data collection infrastructure; difficulty tracking patient records; poor monitoring of disease outcomes	Contact with patients; storage and retention of patient data	Systems to generate and locate patient data are helpful. These systems may aid patients in keeping appointments.	Access: reminder letters, calls, and texts help reach patients. Coverage: telemedicine consults may help NPHWs treat more conditions. Quality: data tracking systems improve care continuity. Safety: patient and disease surveillance may minimize errors.
Medication access	Consistent medication availability; supply chain management staff; compensation of supply, transport costs for medication	Medications and supplies out of stock; staff unfamiliarity with medication availability, proper usage	Supply chains or access pathways; NPHW capacity to use or prescribe medication	Consistent medication and supply chains aid care. Donor support for supply chain logistics can boost consistency. Retaining logistical staff to oversee process may also help.	Access: strong supply chains help patients consistently obtain medications. Coverage: broader formulary allows greater breadth of care. Quality: adequate medications, supplies help adhere to latest care guidelines. Safety: reliable suppliers ensure safe medications.
Financing	Performance-based compensation; donor awareness of local needs	Lack of monetary performance incentives; low clinician pay; underfinance of patient care infrastructure	Supply-side issues (eg, program funding, NPHW salary); demand-side issues (eg, financing patient access)	Clinicians should be adequately compensated. Pay-for-performance models help. Sufficient investment in care delivery system also key.	Access: stronger care infrastructure (and insurance schemes) help patients reach care. Coverage: donor awareness of local disease burden helps finance relevant care packages. Quality: pay-for-performance boosts level of care provided. Safety: adequate funding ensures safe, functional care infrastructure.

Abbreviation: NPHW, nonphysician health worker.

Results varied in scope and content across building blocks, and the diseases discussed in the studies were heterogeneous (Table 2; eTable and eReferences in the Supplement). There were a total of 174 barriers and 170 facilitators. Among both barriers and facilitators, service delivery (69 barriers and 54 facilitators) and health workforce (46 barriers and 62 facilitators) factors appeared most commonly, with governance (17 barriers and 23 facilitators), information systems (12 barriers and 19 facilitators), medication access (12 barriers and 7 facilitators), and financing (13 barriers and 8 facilitators) factors arising intermittently. Among the NCDs covered within these 15 articles, cardiovascular conditions and mental illness were the most common (3 studies each); there were 2 studies pertaining to multiple NCDs, 2 pertaining to diabetes, and only 1 pertaining to sexual violence. Most systematic reviews were robust, scoring between 5 and 14 on an A Measurement Tool to Assess Systematic Reviews–2 scale of 13 to 16 items (Table 3). Among the 71 cross-referenced articles, the diseases treated and delivery context were heterogeneous, with neither grossly associated with specific care barriers or facilitators (Table 2; eTable and eReferences in the Supplement).

**Table 3.** A Measurement Tool to Assess Systematic Reviews–2 Evaluation of Systematic Reviews

Source	PICO Use	Protocol A Priori	Study Design Selection	Robust Search Strategy	Duplicate Study Selection	Duplicate Data Extraction	List of Excluded Studies	Detail Given	RoB Assessed	Funders Listed	Sound Meta-analysis Method	RoB Noted in Meta-analysis	RoB Impact Explored	Heterogeneity	Publication Bias	COI	Final Score^a^
Mutamba et al,^42^ 2013	Yes	Yes	Yes	Yes	No	No	Yes	Yes	Yes	No	NA	NA	Yes	Yes	NA	Yes	10/13
Joshi et al,^10^ 2014	No	Yes	No	Yes	Yes	Yes	Yes	Yes	No	No	NA	NA	No	Yes	NA	Yes	7/13
Ogedegbe et al,^45^ 2014	Yes	Yes	No	Yes	Yes	Yes	Yes	Yes	Yes	No	NA	NA	No	No	NA	Yes	8/13
Khetan et al,^40^ 2017	No	Yes	No	Yes	No	No	Yes	Yes	No	No	NA	NA	No	Yes	NA	Yes	5/13
Jeet et al,^49^ 2017	Yes	Yes	No	Yes	Yes	Yes	Yes	Yes	Yes	No	Yes	Yes	Yes	Yes	Yes	Yes	14/16
Schneider et al,^9^ 2016	No	Yes	Yes	Yes	Yes	Yes	Yes	Yes	No	No	NA	NA	No	No	NA	Yes	7/13
Padmanathan et al,^41^ 2013	No	Yes	No	Yes	No	No	Yes	Yes	Yes	No	NA	NA	Yes	No	NA	No	6/13
Abdel-All et al,^44^ 2017	No	Yes	No	Yes	Yes	Yes	Yes	Yes	Yes	No	NA	NA	No	No	NA	Yes	8/13
Seidman et al,^48^ 2017	No	Yes	No	Yes	Yes	No	Yes	Yes	No	No	NA	NA	Yes	No	NA	Yes	7/13
Chowdhary et al,^43^ 2014	Yes	Yes	No	Yes	No	Yes	Yes	Yes	No	No	NA	NA	No	No	NA	No	6/13
Barnett et al,^39^ 2017	Yes	Yes	Yes	Yes	Yes	Yes	Yes	Yes	No	No	NA	NA	No	Yes	NA	Yes	10/13
Hill et al,^47^ 2017	Yes	Yes	Yes	Yes	Yes	Yes	Yes	Yes	No	No	NA	NA	No	No	NA	Yes	9/13
Alaofè et al,^50^ 2017	No	Yes	No	Yes	Yes	Yes	Yes	Yes	Yes	No	NA	NA	No	Yes	NA	Yes	8/13
Gatuguta et al,^38^ 2017	No	Yes	Yes	Yes	No	No	Yes	No	No	No	NA	NA	No	No	NA	Yes	5/13
Javadi et al,^46^ 2017	No	Yes	No	Yes	Yes	Yes	Yes	Yes	Yes	No	NA	NA	No	No	NA	Yes	8/13

Abbreviations: COI, conflict of interest; NA, not applicable; PICO, population studied, intervention performed, comparison group, and outcome; RoB, risk of bias.

^a^ A Measurement Tool to Assess Systematic Reviews–2 has a scale of 13 to 16 items.

### Service Delivery

Three themes emerged among service delivery barriers: logistics, infrastructure, and stigma. Logistical problems included patient difficulty reaching clinics, and, conversely, health worker difficulties reaching patients’ homes.^38,39,40,52,53,54,55,56,57^ Weak care infrastructure caused crowding and lack of privacy,^41,58,59,60^ increasing the wait times for care.^61^ Care delivery barriers included small scale of care,^42^ difficulty scaling services up,^43^ limited curative care,^62,63^ lack of referral systems for care,^64^ and poor integration across care components.^65,66,67^ The stigma of seeking care, especially for mental illness and obesity, was a cultural barrier.^39,43,44,68^ Patient literacy also sometimes hampered care,^53,69^ as did skepticism of treatment plans^56,60,70,71^ and gender-related barriers.^72^

Conversely, delivery facilitators involved creative solutions to logistical barriers and care sensitive to community needs. These strategies included home-based or home-adjacent care,^42,43,45,73^ integrated care across conditions,^72,74,75,76^ use of consistent care protocols,^77,78^ and programs to track and refer patients, including telemedicine.^67,79^ Nimgaonkar et al^70^ described a mental health intervention within a village health worker initiative in India and found that integrating these 2 programs facilitated reaching patients and decreased stigma. Culturally applicable health education was also helpful,^40,43,45,80^ as was community engagement and embeddedness.^46,81^ Abas et al,^82^ offering problem-solving therapy for depression in Zimbabwe, found that employing female health workers of the same socioeconomic status as their patients aided care delivery.^46^

### Health Workforce

Workforce barriers comprised gaps between NPHWs’ capacity to perform key tasks and their supervisors’ expectations and support for them to do so. Workers’ skills and training were often insufficient.^38,40,46,58,69,83,84,85^ Furthermore, supervisors deployed NPHWs ineffectively to use these skills because of unclear job roles,^60,86,87^ excessive workload,^38,60^ and weak or adversarial relationships with other workers.^10,41,62^ Third, weak oversight^65,72,73,74,86,87^ hampered workers’ ability to meet expectations. Staff turnover also hampered care.^8,10,88^

Workforce facilitators addressed the aforementioned gaps but also aided in the judicious selection of workers. Nonphysician health workers who were recruited from the community served, or who were aware of its languages and customs, were an asset.^39,42,43,46,47,70,82^ Also helpful was rigorous, locally relevant training, including care algorithms^10,40,44,47,72,76,77,89^ and close collaboration with other health workers.^10,39,41,45,47,70,77,80,89^ Gaziano et al^12^ evaluated CHWs’ ability to screen for cardiovascular disease risk in Guatemala, Mexico, and Bangladesh and found that careful staff selection, training in local languages, and the use of simple care records facilitated success. Programs for epilepsy care in Kenya^77^ and postpartum depression in Pakistan^89^ also praised detailed care protocols and close supervision, respectively.

### Governance

Governance barriers pertained to the insufficient authority of NPHWs to treat NCDs resulting from the lack of political will to authorize them and the consequent inability to assume key roles. In addition to weak oversight by supervisors, NPHWs reported poor communication with clinical directors,^46^ policy makers,^90^ and other overseers.^84^ In the absence of implementation science data,^8,72^ NPHWs faced mistrust regarding new roles, limiting their scope of care.^40^ These problems were compounded by ambiguity regarding NPHWs’ job roles^38,40^ and limitations on what care nonphysicians could provide,^63^ especially regarding prescribing medications.^10,41,45^ Weak care monitoring^64,91^ and backup support^45^ also arose, as did structural political factors, such as deliberate corruption of purpose,^62^ lack of governmental coordination,^90^ or active deprioritization by the ministry of health,^92^ hindering the distribution of personnel and resources.^62^

However, initiatives providing NPHWs a clear mandate and scope of work integrated within existing health care infrastructure^93^ aided care delivery,^43,46^ especially when policy makers and community officials actively assisted the NPHWs’ work and that of their supervisors^62,76,86,90,94^ and made express commitments to equitable care access.^46^ An evaluation of CHWs’ effectiveness in treating hypertension and diabetes in Iran^94^ noted that their impact was substantially greater for diabetes than hypertension because of Iran’s codification of their role in diabetes care.

### Information Systems

Information system barriers comprised 2 categories: difficulty tracking patients and storing their data for longitudinal care. Nonphysician health workers struggled to record clinical encounters^40,71,73,74^; when present, information storage was inefficient.^8,40,70^ Contacting patients by telephone was also sometimes difficult.^58^ However, strategies to overcome these barriers were diverse and creative. A hypertension and diabetes program in Cameroon sent reminder letters to patients^95^; other programs used electronic or telephone systems for patient tracking,^46,90^ program eligibility and other screening,^96,97^ and surveillance and intervention planning.^8,77,78^ Some programs used telemedicine to provide NPHW oversight^98^ or even remote clinical encounters.^79,92^

### Medication Access

Inconsistent access to medication hampered NPHW care, whereas reliable access promoted it. Many studies^8,10,16,46,62,70,71,76,99^ noted disruptions in medication or equipment as a challenge. Even when present, medications were sometimes expensive,^55^ and NPHWs were not always trained on how to use them.^100^ Interventions that leveraged consistent, inexpensive medication access were invaluable,^8,10,46,77,78^ but uncommon. Joshi et al,^78^ in discussing strategies to strengthen nonphysician cardiovascular disease care in rural China, noted that cost-effective drug distribution plans were helpful, as was the use of a single-tablet, multidrug polypill. A nurse-led hypertension treatment program in Cameroon^101^ also benefited from locally available medication.

### Financing

Finance barriers fell into 2 categories: supply-side issues, such as lack of program funding^41,46,64^ and consequent poor pay for NPHWs,^44,88^ and mismanagement of resources, including poor performance incentives,^74^ lack of long-term planning,^91^ and overuse of out-of-pocket models for reimbursement (instead of insurance subsidies). The latter issue decreased patients’ demand for care^102^ and encouraged curative care in lieu of preventive medicine.^62^ Financial facilitators of care, conversely, included not only adequate program and salary funds^8,62^ but also social protection schemes to encourage patients to access care.^97^ One study,^91^ evaluating the integration of mental health care into CHW-led primary care in Kenya, noted that donor awareness of local needs optimized allocation of resources.

## Discussion

We undertook a systematic review of health system factors that support or impair NPHW-led interventions to control NCDs in LMICs. Issues involving service delivery, the health workforce, and program governance encompassed the most diverse themes, ranging from logistical matters (eg, the distribution of workers), to structural issues of oversight, to cultural ones (eg, stigma). However, in all 6 domains, most themes pertained to either sufficient quantity of resources (eg, size of clinics or procurement of medications) or their effective stewardship (eg, authorizing NPHWs to use these medicines). Actionable lessons emerged across each block, as outlined here:

1. Service delivery: Provide protocol-based NPHW care that is community based and culturally sensitive to the community served, with physical infrastructure to allow access and referral based on robust physician backup systems.
2. Workforce: Select qualified NPHWs responsive to and embedded within communities, and provide rigorous training to workers and support from supervisors.
3. Governance: Provide detailed, feasible work expectations, with explicit support from clinical leadership and policy makers. Grant authority for NPHWs to perform appropriate clinical duties, including prescribing medication.
4. Information systems: Furnish electronic systems to allow NPHWs to remotely contact patients, follow their clinical data, and assess their eligibility for health interventions.
5. Medications: Provide a consistent supply chain of essential drugs, an efficient system for their distribution to patients, and training on appropriate use.
6. Financing: Provide adequate funds for essential program supplies, pay NPHWs fairly and consistently, and minimize patient out-of-pocket costs.

**Figure 2.**
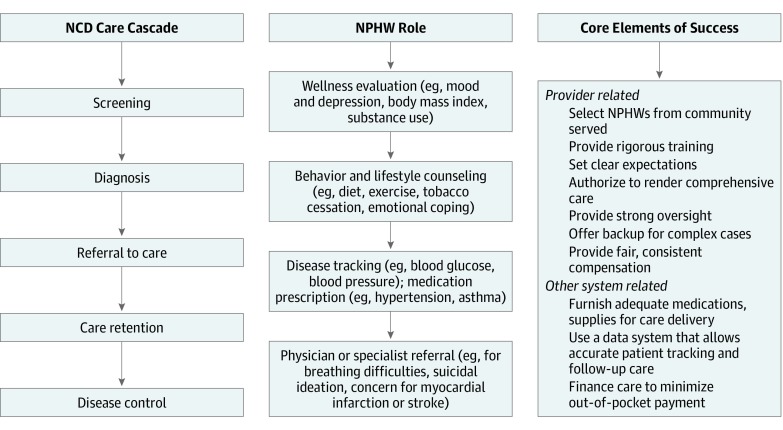
Nonphysician Health Workers (NPHWs) in the Noncommunicable Disease (NCD) Care Cascade Chart shows steps and NPHW role in the NCD cascade.

These findings also inform other frameworks specific to the performance of nonphysicians. The US-based CHW Core Consensus Project,^103^ for example, uses a framework asking how CHWs’ roles have evolved, what roles they currently play, and what skills they need to fill these roles. We found a substantial increase in NPHW-led NCD care models in recent years in all aspects of the NCD care cascade (Figure 2); more broadly, we found that NPHWs should be selected from within the communities served, rigorously trained, and given authority and material support to do their work. Similarly, Palazuelos et al^104^ designed a 5-point framework to appraise the resources CHWs need in LMIC settings: supervision, partnership, incentives, choice (recruitment), and education. These concepts, too, parallel the need for NPHW oversight, role definition, remuneration, selection, and training, respectively, as described already.

### Literature Gaps

Few studies devised or evaluated process measures that could reveal whether and why their interventions operated consistently. For example, tracking how health care workers order key medications, when the medications arrive at clinics, and how often patients actually receive them could address the problem of medications being out of stock. Cost-effectiveness data were also rare,^15,58,63,102,104^ despite their implications for health policy. Furthermore, studies’ use of novel technologies and governance models raised unanswered questions, such as how best to use mobile telephones for decision support or how to implement performance-based compensation. In addition, although adequate compensation and supervision were crucial to NPHWs’ day-to-day performance, strategies for workers’ retention and promotion did not arise.

### Health System Framework Challenges

The WHO’s building block framework did not accommodate all care barriers and facilitators described^105,106^ because some factors operate outside the health system. For example, some patient access barriers, such as community suspicion of government and inclement weather and roads, derive from factors not directly tied to health but still speak to themes (ie, social dynamics, climate, and built environment) that are instrumental to NPHW-led care. These political and cultural factors also influence the health system proper—for example, through the power dynamics that dictate how policy makers implement a health intervention, or the social values with which communities receive it (ie, the “software” through which the system’s building-block “hardware” renders care).^107^ This descriptive gap underscores the need for health care frameworks to acknowledge extrasystem barriers and facilitators; integrate health system factors with social, economic, and environmental factors; and use a multidisciplinary approach to NPHW system integration.

Also, some barriers and facilitators fit into multiple blocks.^25,26^ These included NPHW remuneration (workforce vs financing), NPHW team integration (workforce vs governance), and training on medication use (workforce vs medication). Although we categorized these elements by consensus, as described in the Methods section, the ambiguity highlights the interrelatedness of the building blocks. However, the relevance of the exact categorization is debatable, insofar as the blocks are interconnected,^106^ with cross-block innovations required to enable effective NPHW care.^108^

### Policy and Program Implications

Our review suggests both practices and policy ideas to optimize NPHW-led NCD care, which some groups have begun to use. Partners in Health’s Mentorship and Enhanced Supervision for Health Care and Quality Improvement initiative,^109,110^ for example, stipulates that NPHW mentors continuously observe mentees every 4 to 6 weeks using a checklist to ensure quality of care. The Rural Andhra Pradesh Cardiovascular Prevention Study,^111^ a randomized trial of nonphysician hypertension screening and referral in India, benefited from a simple patient evaluation algorithm. The Nigerian Anti-Hypertensive Trial,^112^ a randomized trial of nurse-led hypertension treatment, benefited from nurses’ permission to dispense medications. Unfortunately, despite evidence that such interventions are effective^12^ and culturally acceptable,^82^ improve outcomes,^48^ and control costs,^70,113,114^ few have been scaled into national-level health systems.^102^

These findings are also applicable to high-income countries such as the United States, where physician assistants, nurses, and pharmacists provide a growing fraction of care.^115^In addition, nonphysicians are providing care outreach in novel settings, such as high blood pressure counseling and treatment in barber shops^116^ and churches.^117^ The Robert Wood Johnson foundation recently convened a task force identifying novel examples of global nonphysician care that can be applied in underserved US communities^118^; similarly, an intervention in Indiana is leveraging interventions validated in LMICs to reduce infant mortality.^119^

### Limitations

Our review approach had notable limitations. We did not search databases beyond PubMed to identify systematic reviews, nor did we review articles not cited within the systematic reviews we identified, introducing bias both in our selection of studies and inherent within them (Table 3). Furthermore, although 2 reviewers examined all articles, they split the initial review of these articles, with each half reviewed post hoc by the other. Given the ambiguity and overlap of the WHO’s health system building block classification, including many barriers and facilitators not covered within the 6 blocks, it is possible that we missed or incorrectly categorized pertinent findings, potentially altering the results. Although we detailed the care site and disease treated within each study, we did not quantify such trends within these heterogeneous data. Nonetheless, strengths of this work include its detailed, reproducible systematic review protocol, its review of all classifications with a third coauthor, and its codevelopment of approach with a research librarian.

## Conclusions

Because NCDs are now the leading cause of premature death and disability in low-income regions where physicians remain scarce, we sought to understand how NPHW-led programs for the control of these diseases are aided or impaired by the health systems they inhabit. This study found a small but growing set of studies describing these health system barriers and facilitators, usually incidentally rather than by the studies’ design, but with frequent and consistent messages regarding each of the WHO’s health system building blocks.

Beyond the training, retention, supervision, and deployment of these workers, furnishing strong support systems (supply chains for medications and equipment) and other infrastructure (telephone patient tracking or electronic patient records), as well as adequate program funding, also may facilitate success. In addition, further implementation research—in which these elements are expressly furnished, delivered, and evaluated for delivery and its care impact—will better illuminate how these elements are associated with care delivery. With these data, the effective scale-up of NPHW programs for the leading cause of morbidity and mortality in LMICs may be feasible.

## Notes

Supplement. **eAppendix.** PubMed Search Terms for Study **eTable.** Details of Referenced Studies Citing Barriers and Facilitators to Nonphysician Care **eReferences.**
