# Supplementary material for: Assessment of Barriers and Facilitators to the Delivery of Care for Noncommunicable Diseases by Nonphysician Health Workers in Low- and Middle-Income Countries: A Systematic Review and Qualitative Analysis
Source: JAMA Netw Open. 2019 Dec 2;2(12):e1916545. doi: 10.1001/jamanetworkopen.2019.16545 (PMC6902752; doi:10.1001/jamanetworkopen.2019.16545)
Supplement: Supplement. — eAppendix. PubMed Search Terms for Study eTable. Details of Referenced Studies Citing Barriers and Facilitators to Nonphysician Care eReferences. [file jamanetwopen-2-e1916545-s001.pdf]

Supplementary Online Content

Heller DJ, Kumar A, Kishore SP, Horowitz CR, Joshi R, Vedanthan R. Assessment of barriers and facilitators to the delivery of care for noncommunicable diseases by nonphysician health workers in low- and middle-income countries: a systematic review and qualitative analysis. *JAMA Netw Open*. 2019;2(12):e1916545. doi:10.1001/jamanetworkopen.2019.16545

eAppendix. PubMed Search Terms for Study
eTable. Details of Referenced Studies Citing Barriers and Facilitators to Nonphysician Care
eReferences.

This supplementary material has been provided by the authors to give readers additional information about their work.

© 2019 Heller DJ et al. *JAMA Netw Open.*

eAppendix. PubMed Search Terms for Study

((((((((((((((((("Non-physician health worker") OR "Non physician health worker") OR "Non-physician health workers") OR "Non physician health workers") OR CHW) OR "community health worker") OR "community health workers") OR "non-physician clinician") OR "non-physician clinicians") OR "non-physician clinicians") OR "non physician clinicians") OR "Task shifting") OR "Task-shifting") OR Task shifting) OR "task sharing") OR "task-sharing")) AND ((systematic[sb] OR "review"[Publication Type]))

© 2019 Heller DJ et al. *JAMA Netw Open.*

eTable. Details of Referenced Studies Citing Barriers and Facilitators to Nonphysician Care

Name/Year	Citing Reviews	Disease treated	Barriers	BC	Facilitators	FC	Sex Treated	Delivery Site
**Tripathy et al,[1] 2010**	Mutamba et al,[2] 2013; Chowdhary et al,[3] 2014	Maternal depression	-	-	Availability of referral services and a strong supervisory system; training and support structure	HW	Female	-
**Dias et al,[4] 2008**	Mutamba et al,[2] 2013; Schneider et l,[5] 2016	Dementia	-	-	Locally recruited individual, local materials/resources for training; support and supervision from local psychiatrists	SD	-	Home
**Ali et al,[6] 2003**	Mutamba et al,[2] 2013; Barnett et al,[7] 2017; Javadi et al,[8] 2017	Anxiety; Depression	Stigma of mental health care	SD	-	-	Female	-
**Neuner et al,[9] 2008**	Mutamba et al,[2] 2013; Barnett et al,[7] 2017; Javadi et al,[8] 2017	PTSD	Loss to follow-up (migrant refugees); lack of mental health inclusion in primary care package (SD)	SD	-	-		Home, “quiet site” nearby
**Haines et al,[10] 2007**	Mutamba et al,[2] 2013; Joshi et al,[11] 2014; Schneider et l,[5] 2016	Child survival	Weak health systems; perceived effectiveness of vertical programs; inability to provide simple curative interventions; Perceived superiority of formally trained	SD; HW; M; F; G	focused tasks; community location and infrastructure (closer to health facility); training, supervision, strong community involvement & monitoring to fight against appointment system; educating medical students on importance/handling of	SD; HW;F	-	-

© 2019 Heller DJ et al. *JAMA Netw Open.*

			health personnel and lack of training in supporting CHWs; paternalism/hierarchy coloring relationship of CHWs to health personnel;		CHWs; training CHWs in basic vitals signs and considering local sx terminology and illness beliefs; multi-faceted interventions vs single component or written guidelines; Supervision - checklist, two-way info flow, role model, peer support; problem-solving approach			
**Feksi et al,[12] 1991**	Joshi et al,[11] 2014	Epilepsy	-	-	treatment protocols for non-medical personnel; health worker support, psychiatrist supervision; appropriate technology; data collection done by specialists for Quality control, even if service delivery is done by NPHW; guaranteed drug supply	HW;IS ; MA	-	Clinic
**Coleman et al,[13] 1998**	Joshi et al,[11] 2014	HTN, Diabetes, Asthma	-	-	practical, step-wise protocols for dx and tx; simplifying drug tx + rationalizing lab use	SD	-	Clinic
**Joshi et al,[14] 2012**	Joshi et al,[11] 2014; Khetan et al,[15] 2017; Jeet et al,[16] 2017	CVD risk (teaching)	-	-	NPHW ability to rx simplified polypill (G); simple algorithms (HW)	G; HW	-	Village Health Centre
**Labhardt et al,[17] 2010**	Joshi et al,[11] 2014	HTN, Diabetes	No equipment or drugs; Lack of requisite knowledge	MA; HW;	-	-	-	Clinic

			for HCWs; Frequent reallocation of staff					
**Kengne et al,[18] 2009**	Joshi et al,[11] 2014	Diabetes, HTN	--	-	Nurses given Rx power; Locally available meds; Protocol-driven NPHW care	HW; MD; SD	-	Clinic
**Labhardt et al,[19] 2011**	Joshi et al,[11] 2014	HTN, Diabetes	L/R barriers: Drug prices, transportation costs	SD; M	Linkage/retention facilitators: treatment contracts, financial incentives, reminder letters	SD; I	-	Clinic
**Pisani et al,[20] 2006**	Joshi et al,[11] 2014	Breast Cancer	Lack of training and quality control; culturally-related health beliefs	HW; SD/O (?)	-	-	Female	Health Centre
**Adams et al,[21] 2012**	Joshi et al,[11] 2014	Depression	disrupted medication supply	MA	-	-	-	Clinic
**Bhanbhro et al,[22] 2011**	Joshi et al,[11] 2014	All (NPHWs prescribed, review)	-	-	NMP Legal Frameworks; patient group directions or standing orders for CHWs for EDL/Immunizations	G	-	-
**Mdege et al,[23] 2013**	Joshi et al,[11] 2014	HIV	Lack of previous curative CHW services; National statutory requirements or policies that stipulate specific tasks for different cadres	SD; G	Robust evaluation framework; adequate preparation, training, support, supervision; strong govt leadership, political commitment/support, broad-based partnership, open dialogue/communication, supportive policy/social environment; public	SD; HW; G	-	Clinics; Health Centers

© 2019 Heller DJ et al. *JAMA Netw Open.*

					education campaigns; local/external resources; Maintain quality of care standards; implementation guidelines from previous studies (intervention components, environment, etc); standards for reporting such guidelines			
**Rahman et al,[24] 2008**	Joshi et al,[11] 2014; Schneider et l,[5] 2016, Chowdhary et al,[3] 2014; Barnett et al,[7] 2017; Javadi et al,[8] 2017; Padmanathan et al,[25] 2013	Perinatal Depression	-	-	New tasks should not be extra burden/relevant to everyday work; Monthly half-day group supervision provided by specialists; reliable training and supervision	SD; HW	Female	-
**Patel et al,[26] 2011**	Joshi et al,[11] 2014; Javadi et al,[8] 2017	Depress-ion, Anxiety	Medication shortages	M	-	-	-	Clinics/ Facilities
**Petersen et al,[27] 2012**	Joshi et al,[11] 2014; Barnett et al,[7] 2017	Depression	-	-	Trained CHWs under supervision of mental health specialist	HW	-	Clinic
**Joshi et al,[28] 2008**	Joshi et al,[11] 2014	CVD (risk factors; policy paper)	-	-	Screening tools/tx packages; health record systems/electronic decision support; cost-effective drug distribution; health surveillance; low-dose polypill for ease of rx	SD; I; MA	-	-
**Lewin et al,[29] 2010**	Joshi et al,[11] 2014;	Maternal health, child	Availability of routinely collected	I	Considering similar settings for implementation of	SD; F; MA	-	-

© 2019 Heller DJ et al. *JAMA Netw Open.*

	Schneider et l,[5] 2016	health, infectious diseases	data for targeting interventions		LHW programs as research is conducted; mechanisms for compensation/remuneration ; Adequate drugs/supplies + referral capacity; consideration of baseline incidence/prevalence of a targeted disease			
**Mendis et al,[30] 2010**	Ogedegbe et al,[31] 2014; Jeet et al,[16] 2017	CVD risk (HTN, behavior)	Lack of robust data collection (IS)	I	Use of simple protocols for assessment, management (treatment + counseling)	HW	-	Clinics/ Facilities
**Nesari et al,[32] 2010**	Ogedegbe et al,[31] 2014	Diabetes	-	-	Nurse can adjust medication via phone but wholsesale changes made in consultation w endocrinologist	SD	-	Telephone
**Jafar et al,[33] 2009**	Khetan et al,[15] 2017	HTN	-	-	Using only one training session to expedite/facilitate training	HW	-	Home
**Pastakia et al,[34] 2013**	Khetan et al,[15] 2017	Diabetes, HTN	linkage to care hampered by lack of home visit option	SD	-	-	-	Home (screen) Commu-nity (then clinic)
**Denman et al,[35] 2015**	Khetan et al,[15] 2017	Health behavior (diet, exercise)	patient exercise initiative impaired by weather; lack of sidewalks; safety concerns	(O; SD?)	-	-	-	Commu-nity Health Centers
**Farzadfar et al,[36] 2012**	Khetan et al,[15] 2017; Schneider et l,[5] 2016,	Diabetes, HTN	-	-	Density of CHW coverage correlated with success; Designating diabetes as government priority	SD; G	-	Rural “Health House”

© 2019 Heller DJ et al. *JAMA Netw Open.*

	Alaofè et al,[37] 2017; Barnett et al,[7] 2017;				facilitated care			
**Balagopal et al,[38] 2012**	Khetan et al,[15] 2017; Schneider et l,[5] 2016; Alaofè et al,[37] 2017; Hill et al,[39] 2017	Diabetes, HTN (education, control)	-	-	engagement with community; education on services	SD	-	Home (door-to-door)
**Siddiqi et al,[40] 2013**	Khetan et al,[15] 2017	Tobacco	poor quality of underlying care facilities impairs tobacco cessation counseling	SD	-	-	~95% men	Health Centers
**Gaziano et al,[41] 2015**	Khetan et al,[15] 2017		low CHW baseline health literacy	HW	Intensive training in LOCAL language, using simple charts; adequate supervision; selection/recruitment criteria (excluding unqualified persons)	HW	-	Commu-nity sites or Homes (then clinics)
**Jafar et al,[42] 2015**	Jeet et al,[16] 2017	HTN	High OOP payments (lack of insurance/coverage)	F	-	-	-	Home (teaching), Clinic (follow-up care)
**Mash et al,[43] 2014**	Jeet et al,[16] 2017	Diabetes	Lack of space for patient education sessions;<br>Difficulty reaching patients for follow-up by phone	SD; I	-	-	-	Health Centers
**Zhong et al,[44] 2015**	Jeet et al,[16] 2017	Diabetes	Limited human resources/workforce	HW; G; O	Offering care for complaints other than	SD; HW; I	-	"Housing Sites"

			numbers; limited time, materials for training; Providers overly focused on private care (pays better); Weak coordination of care across system; meetings at inconvenient times, places for patients (weather)	(?)	diabetes (improved turnout); Training programs to boost providers' confidence; Phone reminders of meetings			
**Abanilla et al,[45] 2011**	Schneider et l,[5] 2016	CVD Risk (HTN, tobacco, etc.)	lack of secondary referral systems; lack of funds; lack of monitoring and evaluation systems	SD; F; G	-	-	-	Churches; Faith-based Sites
**Tsolekile et al,[46] 2014**	Schneider et l,[5] 2016	NCDs (all)	infrequent home visits; limited provider knowledge	SD; HW	-	-	-	Clinics, homes
**Jenkins et al,[47] 2010**	Schneider et l,[5] 2016	Mental illness	poor distribution of training programs; lack of long-term resource planning; lack of monitoring and evaluation systems	HW; F; G	donor awareness of local needs	F	-	Health Facilities
**Chatterjee et al,[48] 2014**	Schneider et l,[5] 2016; Javadi et al,[8] 2017	Schizo- phrenia	-	-	Caregivers promoted medication adherence	HW	-	Health facility, community sites
**Naved et al,[49] 2009**	Padmanathan et al,[25] 2013	Mental Illness; Intimate Partner	Long wait times	SD	-	-	Women	Clinics

© 2019 Heller DJ et al. *JAMA Netw Open.*

		Violence						
**Balaji et al,[50] 2012**	Padmanathan et al,[25] 2013	Schizophrenia	Patients' limited literacy; patient transport; timing for patients' caretakers	SD	-	-	-	Homes, Clinics
**Chatterjee et al,[51] 2008**	Padmanathan et al,[25] 2013	Mental Illness	timing of appointments to fit patient schedules; delays in traveling to treatment sites	SD	-	-		Clinics; Community Sites
**Pereira et al,[52] 2011**	Padmanathan et al,[25] 2013	Mental Illness	Difficulty contacting patients with reminders; patients' distance traveling to appointments; patient adherence (esp if asymptomatic);<br>Lack of provider engagement/interest in care;<br>Medication costs	SD; HW; M	having a backup care consultant	HW	-	Clinics
**Jordans et al,[53] 2007**	Padmanathan et al,[25] 2013	Mental Illness (Psychosocial Counseling)	Lack of integration with existing system; lack of patient confidentiality;<br>Brief training; lack of supervision	SD; HW	-	-	-	-
**Petersen et al,[54] 2011**	Padmanathan et al,[25] 2013; Javadi et al,[8] 2017	Mental Illness	Medication shortages	M	Integrating with other care; educating community members; decentralizing service delivery;<br>Engaging governing authorities regarding care model	SD; G		
**Chibanda et**	Padmanathan	Depression,	Lack of provider	HW;	Home-based visits/care	SD	-	Clinic

© 2019 Heller DJ et al. *JAMA Netw Open.*

**al,[55] 2011**	et al,[25] 2013	Mental Illness	oversight/supervision ; Weak data recording systems	IS				(Bench); Homes
**Abrahams-Gessel et al,[56] 2015**	Abdel-All et al,[57] 2017	CVD Risk (HTN, tobacco, etc.)	Safety issues for providers/patients; gender barriers to care delivery; Language barriers with training materials; limited supervision; heavy workload; Lack of financial or other worker performance incentives; low pay	SD; HW; F	Longer training sessions; interactive training	HW	-	Communit y sites, Homes
**Nishtar et al,[58] 2007**	Abdel-All et al,[57] 2017; Schneider et l,[5] 2016	CVD Risk (HTN, tobacco, etc.)	Insufficient community engagement; insufficient integration of different program components	SD	-	-	No gender focus, but Lady Health Workers used	Communit y sites, Clinics
**Jafar et al,[59] 2011**	Seidman et al,[60] 2017	HTN	High patient out-of-pocket payments	F	-	-	-	Homes, Clinics
**Puett et al,[61] 2013**	Seidman et al,[60] 2017	Severe acute mal-nutrition	-	-	Adequate CHW training	HW	-	Communit y sites, Homes
**Bolton et al,[62] 2014**	Barnett et al,[7] 2017; Javadi et al,[8] 2017	Depression, PTSD	-	-	Adapting explanatory models to culture; "Step sheets" that helped providers follow care algorithms; High levels of provider oversight	SD; HW	-	Communit y sites, Homes, Clinics

© 2019 Heller DJ et al. *JAMA Netw Open.*

**Murray et al,[63] 2015**	Barnett et al,[7] 2017; Javadi et al,[8] 2017	PTSD, emotional trauma	-	-	high workload; poor staff retention; poor provider pay	HW; F	-	Community sites, Homes
**Murray et al,[64] 2013**	Barnett et al,[7] 2017	PTSD, emotional trauma	-	-	Stigma of mental health care; high patient demand (crowding); flooding and long travel times (access barrier)	SD	-	Community sites, Clinics
**Nadkarni et al,[65] 2015**	Barnett et al,[7] 2017	Harmful drinking	-	-	Patient travel distance; patient skepticism of therapy (versus medication); limited patient time	SD	-	Primary Health Centers (Clinics)
**Nimgaonkar et al,[66] 2015**	Barnett et al,[7] 2017; Javadi et al,[8] 2017	Mental illness (general)	Stigma of mental health care; superstition about treatment; Difficulty classifying diseases accurately with existing record systems; Medication shortages	SD; I; M	Integration of mental and non-mental illness care; decentralizing service delivery; Culturally knowledgeable workers	SD; HW	-	Community sites, Clinics
**Mash et al,[67] 2015**	Alaofè et al,[37] 2017	Diabetes	Lack of space to provide care; difficulty communicating with patients (lack of telephones, etc); Superficial [staff] knowledge base	SD; HW	-	-	-	Clinics
**Micikas et al,[68] 2015**	Alaofè et al,[37] 2017	Diabetes	Limited patient written literacy, requiring teaching materials to be adapted;	SD; HW	-	-	-	Homes, Clinics

© 2019 Heller DJ et al. *JAMA Netw Open.*

			Surveyor bias due to inaequate CHW training					
**Ndou et al,[69] 2013**	Alaofè et al,[37] 2017	HTN, Diabetes	Lack of patient trust; limited patient adherence to regimens;<br>Poor monitoring of patient outcomes;<br>Limited procurement of medications and supplies, leading to short supply	SD; I; M	Too few doctor visits; too little physician oversight	HW	-	Homes, Clinics
**Kohli et al,[70] 2012**	Gatuguta et al,[71] 2017	Intimate Partner/ Gender-based Violence	Unsafe provider travel at night; patients not feeling ill (hampered adherence); limited collaboration with existing care groups;<br>Limited systems for patient data tracking	SD; I	-	-	Women	Mobile, Stationary Clinics
**Tanabe et al,[72] 2013**	Gatuguta et al,[71] 2017	Sexual assault	Limited time availability from birth attendants	HW	-	-	Women (chiefly)	Communit y sites
**Abas et al,[73] 2016**	Javadi et al,[8] 2017	Depression, Mental Illness	Weak payment mechanism for providers; high costs for patients	F	Same SES of provider and patient facilitated connection; income generation plan helped recruit patients	SD	-	Clinic (Bench); Homes
**Agyapong et al,[74] 2015**	Javadi et al,[8] 2017	Mental Illness	Poor clarity on role/task; weak oversight;<br>Limited documentation	HW; I	-	-	-	Homes, Clinics
**Agyapong**	Javadi et al,[8]		Weak oversight; lack	HW	Support/collaboration with	HW; G	-	-

© 2019 Heller DJ et al. *JAMA Netw Open.*

**et al,[75] 2015**	2017		of training; unclear role;		psychiatrists/local healers (when available); Engagement with policy stakeholders			
**Agyapong et al,[76] 2016**	Javadi et al,[8] 2017	Mental Illness	Weak oversight; lack of prescribing knowledge/clarity; High cost for patients; Poor connection with policymakers re: care services	HW; F; G	Mobile technology for patient contact; Engagement with policy stakeholders (when this occurred)	I; G	-	-
**Hung et al,[77] 2014**	Javadi et al,[8] 2017	Postpartum Depression	Heavy workloads	HW	Screening technology for eligibility	I	Women	Homes, Clinics
**Larson-Stoa et al,[78] 2015**	Javadi et al,[8] 2017	Depression, Anxiety (post-torture)	Limited financing for full care coverage	F	-	-	-	-
**Lorenzo et al,[79] 2015**	Javadi et al,[8] 2017	Disability (mental or physical)	Lack of coordination across care sectors	SD	Referral management systems	SD	-	-
**Magidson et al,[80] 2015**	Javadi et al,[8] 2017	Depression, PTSD (post-torture)	-	-	Telemedicine aided care delivery	-	-	Clinics, Telephone calls
**Mendenhall et al,[81] 2014**	Javadi et al,[8] 2017	Mental Illness	lack of private space for care; patient transport; lack of support infrastructure; patient skepticism of therapy; unclear CHW roles; lack of specialists; high workload; Poor provider pay	SD; HW; F	-	-	-	-
**Murray et al,[82] 2014**	Javadi et al,[8] 2017	Mental Illness (post-torture)	Patient/provider transportation;	SD; HW;	Apprenticeship training model; "step sheets" to	HW	-	-

© 2019 Heller DJ et al. *JAMA Netw Open.*

			"Personnel problems," perhaps with work consistency?; Lack of stakeholder buy-in	G	follow protocols			
**Padilla et al,[83] 2015**	Javadi et al,[8] 2017	Psychosis	-	-	Local universal health coverage; Illness screening technology	SD; I	-	Homes, Clinics
**Thurman et al,[84] 2014**	Javadi et al,[8] 2017	Mental Illness (in persons with HIV)	-	-	Effective resource allocation across communities	SD	-	Home Visits
**Tomlinson et al,[85] 2011**	Javadi et al,[8] 2017	Alcohol abuse, HIV prevention/care, nutrition, mental illness	-	-	Mobile supervision technology	-	Pregnant women	Home Visits
**Wright et al,[86] 2016**	Javadi et al,[8] 2017	Mental Illness	Training on how to use medication	M	-	-	-	Community sites, Homes, Clinics

BC = Barrier Category; FC = Facilitator Category; SD: Service Delivery; HW: Health Workforce; G: Governance; I: Information Systems; M: Medication Access; F: Financing

eReferences

1. Tripathy P, Nair N, Barnett S, et al. Effect of a participatory intervention with women's groups on birth outcomes and maternal depression in Jharkhand and Orissa, India: a cluster-randomised controlled trial. *Lancet*. 2010;375(9721):1182-1192.
2. Mutamba BB, van Ginneken N, Smith Paintain L, Wandiembe S, Schellenberg D. Roles and effectiveness of lay community health workers in the prevention of mental, neurological and substance use disorders in low and middle income countries: a systematic review. *BMC Health Serv Res*. 2013;13(1):412.

© 2019 Heller DJ et al. *JAMA Netw Open.*

3. Chowdhary N, Sikander S, Atif N, et al. The content and delivery of psychological interventions for perinatal depression by non-specialist health workers in low and middle income countries: a systematic review. *Best Pract Res Clin Obstet Gynecol*. 2014;28(1):113-133.
4. Dias A, Dewey ME, D'Souza J, et al. The effectiveness of a home care program for supporting caregivers of persons with dementia in developing countries: a randomised controlled trial from Goa, India. *PLoS One*. 2008;3(6):e2333.
5. Schneider H, Okello D, Lehmann U. The global pendulum swing towards community health workers in low- and middle-income countries: a scoping review of trends, geographical distribution and programmatic orientations, 2005 to 2014. *Hum Resour Health*. 2016;14(1):65.
6. Ali BS, Rahbar MH, Naeem S, Gul A, Mubeen S, Iqbal A. The effectiveness of counseling on anxiety and depression by minimally trained counselors: a randomized controlled trial. *Am J Psychother*. 2003;57(3):324-336.
7. Barnett ML, Gonzalez A, Miranda J, Chavira DA, Lau AS. Mobilizing community health workers to address mental health disparities for underserved populations: a systematic review. *Adm Policy Ment Health*. 2018;45(2):195-211.
8. Javadi D, Feldhaus I, Mancuso A, Ghaffar A. Applying systems thinking to task shifting for mental health using lay providers: a review of the evidence. *Glob Ment Health (Camb)*. 2017;4:e14.
9. Neuner F, Onyut PL, Ertl V, Odenwald M, Schauer E, Elbert T. Treatment of posttraumatic stress disorder by trained lay counselors in an African refugee settlement: a randomized controlled trial. *J Consult Clin Psychol*. 2008;76(4):686-694.
10. Haines A, Sanders D, Lehmann U, et al. Achieving child survival goals: potential contribution of community health workers. *Lancet*. 2007;369(9579):2121-2131.
11. Joshi R, Alim M, Kengne AP, et al. Task shifting for non-communicable disease management in low and middle income countries: a systematic review. *PLoS One*. 2014;9(8):e103754
12. Feksi AT, Kaamugisha J, Sander JW, Gatiti S, Shorvon SD; ICBERG (International Community-based Epilepsy Research Group). Comprehensive primary health care antiepileptic drug treatment programme in rural and semi-urban Kenya. *Lancet*. 1991;337(8738):406-409.
13. Coleman R, Gill G, Wilkinson D. Noncommunicable disease management in resource-poor settings: a primary care model from rural South Africa. *Bull World Health Organ*. 1998;76(6):633-640.
14. Joshi R, Chow CK, Raju PK, et al. The Rural Andhra Pradesh Cardiovascular Prevention Study (RAPCAPS): a cluster randomized trial. *J Am Coll Cardiol*. 2012;59(13):1188-1196.
15. Khetan AK, Purushothaman R, Chami T, et al. The effectiveness of community health workers in CVD prevention in LMIC. *Glob Heart.* 2017;12(3):233-243.
16. Jeet G, Thakur JS, Prinja S, Singh M. Community health workers for non-communicable diseases prevention and control in developing countries: evidence and implications. *PLoS One*. 2017;12(7):e0180640.
17. Labhardt ND, Balo JR, Ndam M, Grimm JJ, Manga E. Task shifting to non-physician clinicians for integrated management of hypertension and diabetes in rural Cameroon: a programme assessment at two years. *BMC Health Serv Res*. 2010;10(1):339.

© 2019 Heller DJ et al. *JAMA Netw Open.*

18. Kengne AP, Fezeu L, Sobngwi E, et al. Type 2 diabetes management in nurse-led primary healthcare settings in urban and rural Cameroon. *Prim Care Diabetes*. 2009;3(3):181-188.
19. Labhardt ND, Balo JR, Ndam M, Manga E, Stoll B. Improved retention rates with low-cost interventions in hypertension and diabetes management in a rural African environment of nurse-led care: a cluster-randomised trial. *Trop Med Int Health*. 2011;16(10):1276-1284.
20. Pisani P, Parkin DM, Ngelangel C, et al. Outcome of screening by clinical examination of the breast in a trial in the Philippines. *Int J Cancer*. 2006;118(1):149-154.
21. Adams JL, Almond ML, Ringo EJ, Shangali WH, Sikkema KJ. Feasibility of nurse-led antidepressant medication management of depression in an HIV clinic in Tanzania. *Int J Psychiatry Med*. 2012;43(2):105-117.
22. Bhanbhro S, Drennan VM, Grant R, Harris R. Assessing the contribution of prescribing in primary care by nurses and professionals allied to medicine: a systematic review of literature. *BMC Health Serv Res*. 2011;11(1):330.
23. Mdege ND, Chindove S, Ali S. The effectiveness and cost implications of task-shifting in the delivery of antiretroviral therapy to HIV-infected patients: a systematic review. *Health Policy Plan*. 2013;28(3):223-236.
24. Rahman A, Malik A, Sikander S, Roberts C, Creed F. Cognitive behaviour therapy-based intervention by community health workers for mothers with depression and their infants in rural Pakistan: a cluster-randomised controlled trial. *Lancet*. 2008;372(9642):902-909.
25. Padmanathan P, De Silva MJ. The acceptability and feasibility of task-sharing for mental healthcare in low and middle income countries: a systematic review. *Soc Sci Med*. 2013;97:82-86.
26. Patel V, Weiss HA, Chowdhary N, et al. Lay health worker led intervention for depressive and anxiety disorders in India: impact on clinical and disability outcomes over 12 months. *Br J Psychiatry*. 2011;199(6):459-466.
27. Petersen I, Bhana A, Baillie K; MhaPP Research Programme Consortium. The feasibility of adapted group-based interpersonal therapy (IPT) for the treatment of depression by community health workers within the context of task shifting in South Africa. *Community Ment Health J*. 2012;48(3):336-341.
28. Joshi R, Jan S, Wu Y, MacMahon S. Global inequalities in access to cardiovascular health care: our greatest challenge. *J Am Coll Cardiol*. 2008;52(23):1817-1825.
29. Lewin S, Munabi-Babigumira S, Glenton C, et al. Lay health workers in primary and community health care for maternal and child health and the management of infectious diseases. *Cochrane Database Syst Rev*. 2010;(3):CD004015.
30. Mendis S, Johnston SC, Fan W, Oladapo O, Cameron A, Faramawi MF. Cardiovascular risk management and its impact on hypertension control in primary care in low-resource settings: a cluster-randomized trial. *Bull World Health Organ*. 2010;88(6):412-419.
31. Ogedegbe G, Gyamfi J, Plange-Rhule J, et al. Task shifting interventions for cardiovascular risk reduction in low-income and middle-income countries: a systematic review of randomised controlled trials. *BMJ Open*. 2014;4(10):e005983.

© 2019 Heller DJ et al. *JAMA Netw Open.*

32. Nesari M, Zakerimoghadam M, Rajab A, Bassampour S, Faghihzadeh S. Effect of telephone follow-up on adherence to a diabetes therapeutic regimen. *Jpn J Nurs Sci*. 2010;7(2):121-128.
33. Jafar TH, Hatcher J, Poulter N, et al; Hypertension Research Group. Community-based interventions to promote blood pressure control in a developing country: a cluster randomized trial. *Ann Intern Med*. 2009;151(9):593-601.
34. Pastakia SD, Ali SM, Kamano JH, et al. Screening for diabetes and hypertension in a rural low income setting in western Kenya utilizing home-based and community-based strategies. *Global Health*. 2013;9(1):21.
35. Denman CA, Bell ML, Cornejo E, de Zapien JG, Carvajal S, Rosales C. Changes in health behaviors and self-rated health of participants in Meta Salud: a primary prevention intervention of NCD in Mexico. *Glob Heart*. 2015;10(1):55-61.
36. Farzadfar F, Murray CJ, Gakidou E, et al. Effectiveness of diabetes and hypertension management by rural primary health-care workers (Behvarz workers) in Iran: a nationally representative observational study. *Lancet*. 2012;379(9810):47-54.
37. Alaofè H, Asaolu I, Ehiri J, et al. Community health workers in diabetes prevention and management in developing countries. *Ann Glob Health*. 2017;83(3-4):661-675.
38. Balagopal P, Kamalamma N, Patel TG, Misra R. A community-based participatory diabetes prevention and management intervention in rural India using community health workers. *Diabetes Educ*. 2012;38(6):822-834.
39. Hill J, Peer N, Oldenburg B, Kengne AP. Roles, responsibilities and characteristics of lay community health workers involved in diabetes prevention programmes: a systematic review. *PLoS One*. 2017;12(12):e0189069.
40. Siddiqi K, Khan A, Ahmad M, et al. Action to stop smoking in suspected tuberculosis (ASSIST) in Pakistan: a cluster randomized, controlled trial. *Ann Intern Med*. 2013;158(9):667-675.
41. Gaziano TA, Abrahams-Gessel S, Denman CA, et al. An assessment of community health workers' ability to screen for cardiovascular disease risk with a simple, non-invasive risk assessment instrument in Bangladesh, Guatemala, Mexico, and South Africa: an observational study. *Lancet Glob Health*. 2015;3(9):e556-e563.
42. Jafar TH, Jehan I, Liang F, et al. Control of blood pressure and risk attenuation: post trial follow-up of randomized groups. *PLoS One*. 2015;10(11):e0140550.
43. Mash RJ, Rhode H, Zwarenstein M, et al. Effectiveness of a group diabetes education programme in under-served communities in South Africa: a pragmatic cluster randomized controlled trial. *Diabet Med*. 2014;31(8):987-993.
44. Zhong X, Wang Z, Fisher EB, Tanasugarn C. Peer support for diabetes management in primary care and community settings in Anhui Province, China. *Ann Fam Med*. 2015;13(1)(suppl):S50-S58.
45. Abanilla PK, Huang KY, Shinners D, et al. Cardiovascular disease prevention in Ghana: feasibility of a faith-based organizational approach. *Bull World Health Organ.* 2011;89(9):648-656.
46. Tsolekile LP, Puoane T, Schneider H, Levitt NS, Steyn K. The roles of community health workers in management of non-communicable diseases in an urban township. *Afr J Prim Health Care Fam Med*. 2014;6(1):E1-E8.
47. Jenkins R, Kiima D, Okonji M, Njenga F, Kingora J, Lock S. Integration of mental health into primary care and community health working in Kenya: context, rationale, coverage and sustainability. *Ment Health Fam Med.* 2010;7(1):37-47.

© 2019 Heller DJ et al. *JAMA Netw Open.*

48. Chatterjee S, Naik S, John S, et al. Effectiveness of a community-based intervention for people with schizophrenia and their caregivers in India (COPSI): a randomised controlled trial. *Lancet*. 2014;383(9926):1385-1394.
49. Naved RT, Rimi NA, Jahan S, Lindmark G. Paramedic-conducted mental health counselling for abused women in rural Bangladesh: an evaluation from the perspective of participants. *J Health Popul Nutr*. 2009;27(4):477-491.
50. Balaji M, Chatterjee S, Koschorke M, et al. The development of a lay health worker delivered collaborative community based intervention for people with schizophrenia in India. *BMC Health Serv Res.* 2012;12(1):42.
51. Chatterjee S, Chowdhary N, Pednekar S, et al. Integrating evidence-based treatments for common mental disorders in routine primary care: feasibility and acceptability of the MANAS intervention in Goa, India. *World Psychiatry*. 2008;7(1):39-46.
52. Pereira B, Andrew G, Pednekar S, Kirkwood BR, Patel V. The integration of the treatment for common mental disorders in primary care: experiences of health care providers in the MANAS trial in Goa, India. *Int J Ment Health Syst.* 2011;5(1):26.
53. Jordans MJ, Keen AS, Pradhan H, et al. Psychosocial counselling in Nepal: perspectives of counsellors and beneficiaries. *Int J Adv Couns*. 2007;29(1):57-68.
54. Petersen I, Ssebunnya J, Bhana A, Baillie K; MhaPP Research Programme Consortium. Lessons from case studies of integrating mental health into primary health care in South Africa and Uganda. *Int J Ment Health Syst*. 2011;5(1):8.
55. Chibanda D, Mesu P, Kajawu L, Cowan F, Araya R, Abas MA. Problem-solving therapy for depression and common mental disorders in Zimbabwe: piloting a task-shifting primary mental health care intervention in a population with a high prevalence of people living with HIV. *BMC Public Health*. 2011;11(1):828.
56. Abrahams-Gessel S, Denman CA, Montano CM, et al. The training and fieldwork experiences of community health workers conducting population-based, noninvasive screening for CVD in LMIC. *Glob Heart*. 2015;10(1):45-54.
57. Abdel-All M, Putica B, Praveen D, Abimbola S, Joshi R. Effectiveness of community health worker training programmes for cardiovascular disease management in low-income and middle-income countries: a systematic review. *BMJ Open*. 2017;7(11):e015529.
58. Nishtar S, Badar A, Kamal MU, et al. The Heartfile Lodhran CVD prevention project: end of project evaluation. *Promot Educ*. 2007;14(1):17-27.
59. Jafar TH, Islam M, Bux R, et al. Cost-effectiveness of community-based strategies for blood pressure control in a low-income developing country: findings from a cluster-randomized, factorial-controlled trial. *Circulation*. 2011;124(15):1615-1625.
60. Seidman G, Atun R. Does task shifting yield cost savings and improve efficiency for health systems? a systematic review of evidence from low-income and middle-income countries. *Hum Resour Health*. 2017;15(1):29.
61. Puett C, Sadler K, Alderman H, Coates J, Fiedler JL, Myatt M. Cost-effectiveness of the community-based management of severe acute malnutrition by community health workers in southern Bangladesh. *Health Policy Plan*. 2013;28(4):386-399.
62. Bolton P, Bass JK, Zangana GA, et al. A randomized controlled trial of mental health interventions for survivors of systematic violence in Kurdistan, Northern Iraq. *BMC Psychiatry*. 2014;14(1):360.

© 2019 Heller DJ et al. *JAMA Netw Open.*

63. Murray LK, Skavenski S, Kane JC, et al. Effectiveness of trauma-focused cognitive behavioral therapy among trauma-affected children in Lusaka, Zambia: a randomized clinical trial. *JAMA Pediatr*. 2015;169(8):761-769.
64. Murray LK, Familiar I, Skavenski S, et al. An evaluation of trauma focused cognitive behavioral therapy for children in Zambia. *Child Abuse Negl*. 2013;37(12):1175-1185.
65. Nadkarni A, Velleman R, Dabholkar H, et al. The systematic development and pilot randomized evaluation of counselling for alcohol problems, a lay counselor-delivered psychological treatment for harmful drinking in primary care in India: the PREMIUM study. *Alcohol Clin Exp Res*. 2015;39(3):522-531.
66. Nimgaonkar AU, Menon SD. A task shifting mental health program for an impoverished rural Indian community. *Asian J Psychiatr*. 2015;16:41-47.
67. Mash R, Kroukamp R, Gaziano T, Levitt N. Cost-effectiveness of a diabetes group education program delivered by health promoters with a guiding style in underserved communities in Cape Town, South Africa. *Patient Educ Couns*. 2015;98(5):622-626.
68. Micikas M, Foster J, Weis A, et al. A community health worker intervention for diabetes self-management among the Tz'utujil Maya of Guatemala. *Health Promot Pract*. 2015;16(4):601-608.
69. Ndou T, van Zyl G, Hlahane S, Goudge J. A rapid assessment of a community health worker pilot programme to improve the management of hypertension and diabetes in Emfuleni sub-district of Gauteng Province, South Africa. *Glob Health Action*. 2013;6(1):19228.
70. Kohli A, Makambo MT, Ramazani P, et al. A Congolese community-based health program for survivors of sexual violence. *Confl Health*. 2012;6(1):6.
71. Gatuguta A, Katusiime B, Seeley J, Colombini M, Mwanzo I, Devries K. Should community health workers offer support healthcare services to survivors of sexual violence? a systematic review. *BMC Int Health Hum Rights*. 2017;17(1):28.
72. Tanabe M, Robinson K, Lee CI, et al. Piloting community-based medical care for survivors of sexual assault in conflict-affected Karen State of eastern Burma. *Confl Health*. 2013;7(1):12.
73. Abas M, Bowers T, Manda E, et al. "Opening up the mind": problem-solving therapy delivered by female lay health workers to improve access to evidence-based care for depression and other common mental disorders through the Friendship Bench Project in Zimbabwe. *Int J Ment Health Syst*. 2016;10(1):39.
74. Agyapong VI, Osei A, Farren CK, McAuliffe E. Factors influencing the career choice and retention of community mental health workers in Ghana. *Hum Resour Health*. 2015;13(1):56.
75. Agyapong VI, Osei A, Farren CK, Mcauliffe E. Task shifting of mental health care services in Ghana: ease of referral, perception and concerns of stakeholders about quality of care. *Int J Qual Health Care*. 2015;27(5):377-383.
76. Agyapong VI, Farren C, McAuliffe E. Improving Ghana's mental healthcare through task-shifting: psychiatrists and health policy directors perceptions about government's commitment and the role of community mental health workers. *Global Health*. 2016;12(1):57.

© 2019 Heller DJ et al. *JAMA Netw Open.*

77. Hung KJ, Tomlinson M, le Roux IM, Dewing S, Chopra M, Tsai AC. Community-based prenatal screening for postpartum depression in a South African township. *Int J Gynaecol Obstet*. 2014;126(1):74-77.
78. Larson-Stoa D, Jacobs GA, Jonathan A, Poudyal B. Effect of counseling by paraprofessionals on depression, anxiety, somatization, and functioning in Indonesian torture survivors. *Torture*. 2015;25(2):1-11.
79. Lorenzo T, van Pletzen E, Booyens M. Determining the competences of community based workers for disability-inclusive development in rural areas of South Africa, Botswana and Malawi. *Rural Remote Health*. 2015;15(2):2919.
80. Magidson JF, Lejuez CW, Kamal T, et al. Adaptation of community health worker-delivered behavioral activation for torture survivors in Kurdistan, Iraq. *Glob Ment Health (Camb)*. 2015;2(Jan):e24.
81. Mendenhall E, De Silva MJ, Hanlon C, et al. Acceptability and feasibility of using non-specialist health workers to deliver mental health care: stakeholder perceptions from the PRIME district sites in Ethiopia, India, Nepal, South Africa, and Uganda. *Soc Sci Med*. 2014;118:33-42.
82. Murray LK, Dorsey S, Haroz E, et al. A common elements treatment approach for adult mental health problems in low-and middle-income countries. *Cogn Behav Pract*. 2014;21(2):111-123.
83. Padilla E, Molina J, Kamis D, et al. The efficacy of targeted health agents education to reduce the duration of untreated psychosis in a rural population. *Schizophr Res*. 2015;161(2-3):184-187.
84. Thurman TR, Kidman R, Taylor TM. Does investment in home visitors lead to better psychological health for HIV-affected families? results from a quasi-experimental evaluation in South Africa. *AIDS Care*. 2014;26(1)(suppl):S2-S10.
85. Tomlinson M, Doherty T, Jackson D, et al. An effectiveness study of an integrated, community-based package for maternal, newborn, child and HIV care in South Africa: study protocol for a randomized controlled trial. *Trials*. 2011;12(1):236.
86. Wright J, Chiwandira C. Building capacity for community mental health care in rural Malawi: findings from a district-wide task-sharing intervention with village-based health workers. *Int J Soc Psychiatry*. 2016;62(6):589-596.

© 2019 Heller DJ et al. *JAMA Netw Open.*
